# A Review: Bovine Coronavirus Evolution, Molecular Epidemiology, and Genetic Variation

**DOI:** 10.3390/v18080909

**Published:** 2026-08-18

**Authors:** Dong Wang, Wenzheng Zhang, Zheng Nie, Xutian Wang, Jinhui Liu, Yannan Zhang, Yabin Lu, Zhanhai Mai, Xiaodong He, Jianlong Li, Chao Gong, Qingyong Guo

**Affiliations:** 1 College of Veterinary Medicine, Xinjiang Agricultural University, Urumqi 830052, China; xjau-wangdong@foxmail.com (D.W.); 15293814767@163.com (W.Z.); 15136000676@163.com (Z.N.); xvt9981@163.com (X.W.); 17870102312@163.com (J.L.); zynmed@163.com (Y.Z.); lyb4095@163.com (Y.L.); mzh881231@126.com (Z.M.); shouyihexiaodong@sina.com (X.H.); pbb0108@163.com (J.L.); 18328042911@163.com (C.G.); 2 Xinjiang Key Laboratory of New Drug Research and Development for Herbivorous Animals, Urumqi 830052, China; 3 Xinjiang Regional Key Laboratory of Clinical Veterinary Medicine Research, Urumqi 830052, China; 4 Herbivorous Animal Biomedicine and Diagnostic Technology Engineering Research Center, Xinjiang Uygur Autonomous Region, Urumqi 830052, China

**Keywords:** bovine coronavirus, genetic variation, spike protein, phylogenetics, molecular epidemiology, host genetics

## Abstract

Bovine coronavirus (BCoV) is a key pathogen causing calf diarrhea and bovine respiratory diseases, bringing sustained economic losses to the cattle industry. As an RNA virus, BCoV possesses high mutation and recombination capacities, leading to prominent genomic genetic diversity. The genome contains hypervariable and conserved regions, with the S (especially S1), HE and Open Reading Frame (ORF4) genes serving as major variation hotspots linked to viral antigenicity, tissue tropism shift and immune evasion. Host immune pressure drives strong positive selection on S protein antigenic variation. This review discusses existing research limitations and proposes future directions including genomic surveillance, reverse genetics verification and broad-spectrum vaccine development to support BCoV prevention and control.

## 1. Introduction: The Biological Significance of Bovine Coronaviruses and the Importance of Studying Their Variants

Coronaviruses (CoVs) are enveloped viruses with large, complex RNA genomes up to 32 kb in length, encoding 16 non-structural proteins that regulate RNA synthesis and modification. The family Coronaviridae comprises four genera: Alphacoronavirus, Betacoronavirus, Gammacoronavirus, and Deltacoronavirus. These viruses exhibit variable tissue tropism, are capable of crossing species barriers to infect both mammals and birds, and cause diseases with significant differences [1]. Bovine coronavirus (BCoV) belongs to the family Coronaviridae and the genus Betacoronavirus [2]. As a major pathogen responsible for various diseases in cattle, BCoV infection manifests as three distinct clinical syndromes: calf diarrhea (CD), winter dysentery (WD) in adult cattle, and the bovine respiratory disease complex (BRDC) [3,4,5]. In addition, BCoV is commonly detected in healthy cattle [6], with the virus being shed in both feces and nasal secretions. It causes significant economic losses to the global cattle industry. Particularly under intensive farming conditions, BCoV spreads rapidly, resulting in high morbidity and mortality rates, which severely impact cattle production performance and animal welfare. More importantly, the impact of BCoV on herd health is not limited to direct losses during acute outbreaks; it also manifests in the virus’s ability to persist across different ages and production stages, leading to stunted growth, reduced feed efficiency, increased risk of secondary infections, and decreased herd production stability. Because the virus is capable of infecting both the gastrointestinal and respiratory tracts, its prevalence often involves a combination of silent transmission and periodic outbreaks, rendering traditional control strategies that rely on clinical symptoms somewhat lagging. Therefore, re-examining BCoV from the perspective of genetic variation and evolutionary adaptation not only holds significant theoretical value but also directly relates to the optimization of disease control systems and the improvement of production efficiency in the cattle industry.

With the rapid advancement of molecular biology and genomics technologies, research on bovine coronavirus (BCoV) has gradually shifted from traditional pathogen identification and pathological observation to the analysis of its genetic variation mechanisms and evolutionary patterns. As a single-stranded positive-sense RNA virus, BCoV exhibits a high intrinsic mutation rate during replication due to the lack of proofreading function in its RNA-dependent RNA polymerase (RdRp). Additionally, frequent homologous recombination occurs, and together these factors drive the evolution of its genomic diversity. These variations are not randomly distributed but exhibit significant regional heterogeneity across the genome. Among these, the gene encoding the spike protein (Spike, S)—particularly the S1 subunit—as well as the hemagglutinin-esterase (HE) gene and genes for accessory proteins such as ORF4, constitute the “hotspot regions” where mutations are most concentrated. Variations in the S gene not only directly affect the virus’s antigenicity but are also closely associated with changes in its tissue tropism, such as the shift from the gastrointestinal tract to the respiratory tract; mutations in the HE gene may alter the virus’s receptor-binding ability and host adaptability. These variations have also been corroborated at the molecular epidemiological level. Phylogenetic analysis indicates that global BCoV strains are primarily classified into the classical (European) and American wild ruminant (American) lineages, with their geographic distribution patterns closely aligning with international cattle trade routes. Furthermore, the gradual substitution of key amino acid sites on the spike protein has become an important molecular marker for distinguishing between different lineages.

**Figure 1:**
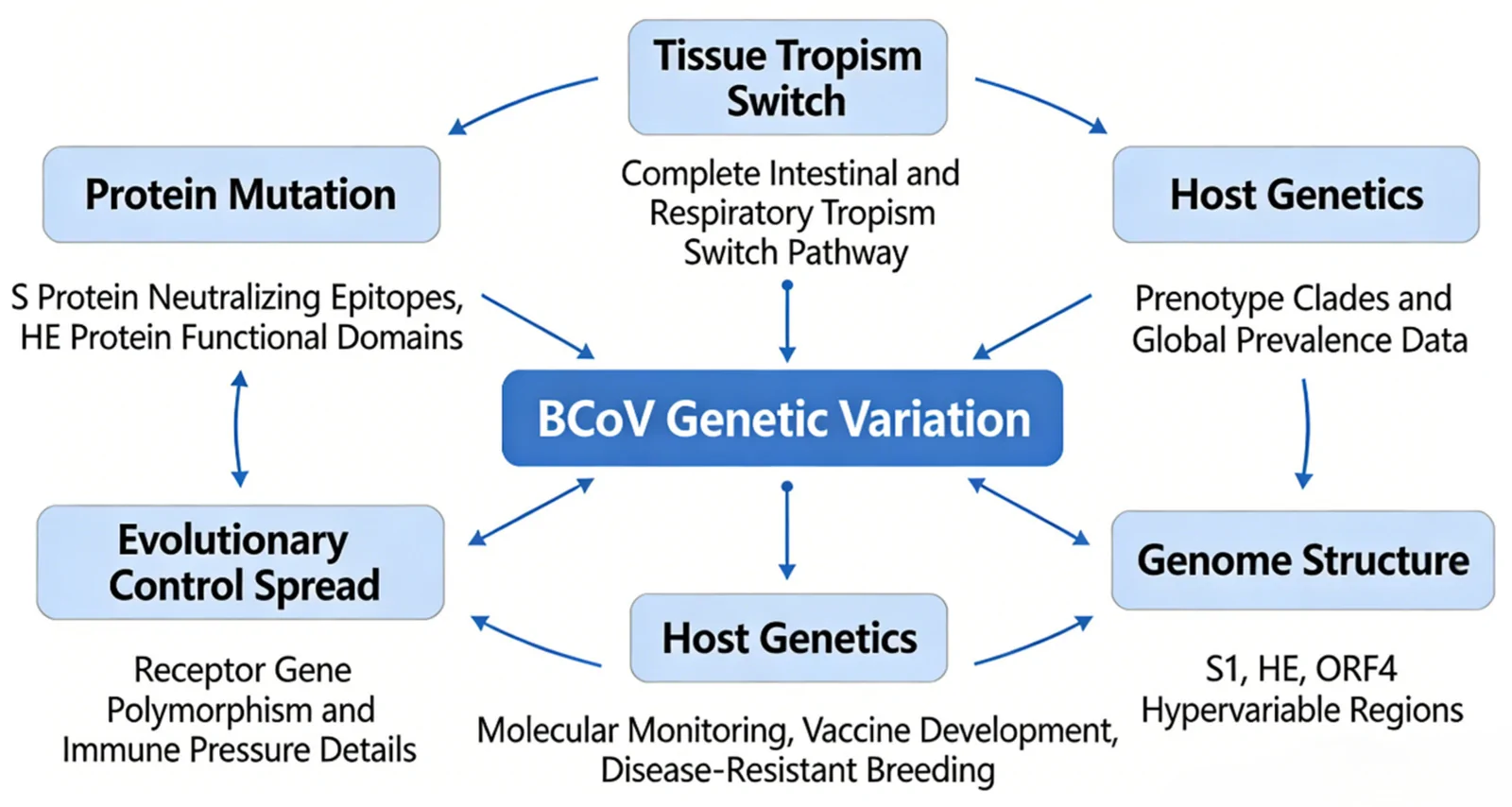
Research hotspots and logical framework of bovine coronavirus genetic variation. The figure shows the core mutation hotspots of the genome, the phenotypic effects of variation (antigenicity change, tissue tropism shift), the driving role of host genetics and immune pressure, and the final application directions of molecular monitoring and vaccine development.

Viral evolution does not occur in isolation but is a dynamic process driven by the combined effects of host immune pressure and the virus’s own replication mechanisms. During transmission, BCoV is continuously subjected to positive selection pressure, particularly in highly immunogenic regions such as the S protein; antigenic variation has become a key strategy for the virus to evade the host’s neutralizing antibody response. In addition to viral factors, the genetic background of the host (cattle) may also modulate the infection process; in particular, genetic polymorphisms in genes encoding viral receptors (such as sialic acid receptors) may influence viral entry efficiency, tissue distribution, and disease severity. In summary, BCoV variation results from the interplay of multifaceted factors. The core research hotspots and logical relationships of BCoV genetic variation are summarized in Figure 1. A thorough analysis of these mechanisms not only helps elucidate the virus’s evolutionary patterns but also provides crucial guidance for optimizing diagnostic methods, iteratively updating vaccines, and scientifically formulating prevention and control strategies. Currently, the prevention and control of BCoV in cattle production mainly relies on commercial inactivated and attenuated vaccines developed from classical strains. However, continuous viral antigenic variation and regional differences in prevalent genotypes have raised concerns about the cross-protective efficacy of existing vaccines against field strains. Therefore, this review will systematically summarize recent research advances in areas such as genomic structure, key protein mutations, associations with clinical manifestations, phylogenetic characteristics, transmission dynamics, and host interactions, with the aim of providing a theoretical basis for the global monitoring, vaccine optimization and precise prevention and control of BCoV.

## 2. Overview of the BCoV Genomic Structure and Mutation Hotspots

Bovine coronavirus (BCoV) is taxonomically classified under the order Nidovirales, family Coronaviridae, and genus Betacoronavirus, specifically as Betacoronavirus type 1 [7]. Its genome consists of a single-stranded positive-sense RNA approximately 31 kb in length, making it one of the largest known RNA viral genomes; it contains 13 open reading frames as well as 5′ and 3′ untranslated regions [8]. The 5′-terminal two-thirds of the genome encode ORF1a and ORF1b. ORF1a encodes the polyprotein pp1a, which is processed via ribosomal frameshifting into pp1ab and subsequently proteolytically cleaved into multiple nonstructural proteins (NSPs) [9]. The 3′-terminal region encodes five major structural proteins: the spike (S) protein, the membrane (M) protein, the hemagglutinin esterase (HE) protein, the envelope (E) protein, and the nucleocapsid (N) protein [10]. From a functional perspective, the front end of the BCoV genome is primarily responsible for replication and transcription-related processes, while the rear end concentrates on encoding structural and accessory proteins involved in host recognition, viral assembly, and release. This organizational structure ensures that the virus possesses a relatively stable replication framework while retaining the ability to respond rapidly to external selective pressures. Generally speaking, non-structural protein regions involved in basic life processes are subject to strong purifying selection and are therefore relatively conserved; in contrast, gene regions directly exposed to host immune pressure or involved in receptor recognition are more prone to accumulating mutations. Consequently, although the overall genetic diversity of BCoV is not extremely high, local mutations in key surface proteins and accessory proteins are still sufficient to elicit significant biological effects.

**Figure 2:**
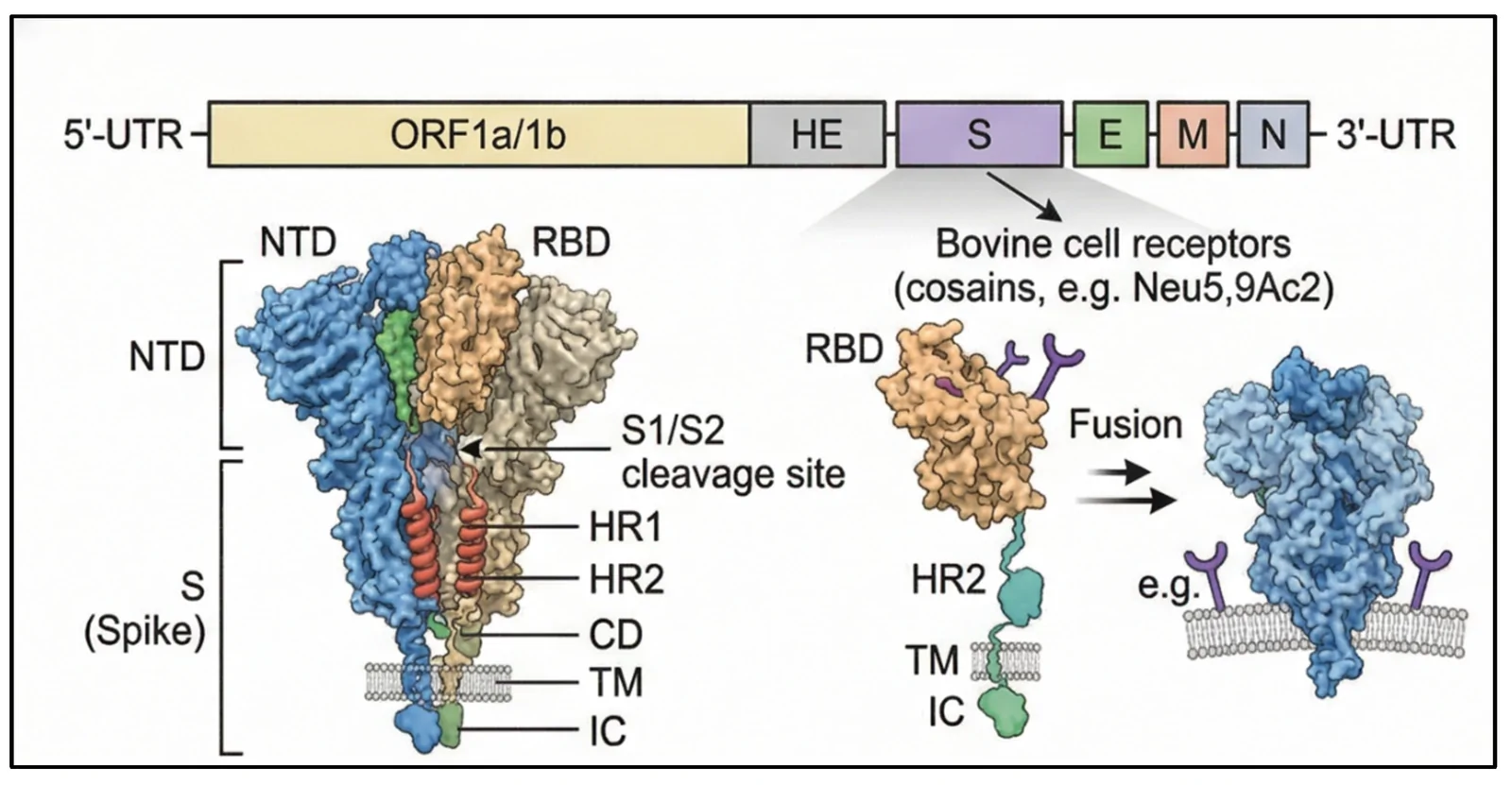
Genome organization of BCoV and structural domain composition of spike protein. The upper part shows the full-length genome structure and open reading frame distribution; the lower part shows the S protein structure, including NTD, RBD in the S1 subunit, the S1/S2 cleavage site, and the Heptad Repeat 1 (HR1), Heptad Repeat 2(HR2), Transmembrane domain(TM) and Intracellular domain(IC) domains in the S2 subunit.

Compared with other members of this family, BCoV exhibits relatively low overall genetic diversity. The nucleotide diversity of its whole genome is about 0.5–2.0%, and the evolutionary rate is estimated to be approximately 10^−4^ to 10^−3^ substitutions per site per year, which is at a medium-low level among coronaviruses [11,12,13]. A quantitative comparison of genetic diversity and evolutionary rate among typical coronaviruses is shown in Table 1; however, coronaviruses belong to a rapidly evolving viral group characterized by the continuous emergence of new variants [11]. Among these, the nucleocapsid (N) protein is highly conserved across different BCoV strains and is commonly used as a target for molecular diagnostics; the spike (S) protein consists of the S1 subunit, responsible for receptor binding, and the S2 subunit, which mediates membrane fusion. It can induce the production of neutralizing antibodies in the host, making it an ideal antigen source for the development of immunodiagnostic assays [14,15]. The hemagglutinin esterase (HE) protein acts as a receptor-cleaving enzyme (esterase) to reverse hemagglutination. The nucleocapsid (N) protein is located within the viral envelope and binds to viral RNA; the membrane (M) protein spans the viral envelope; and the S protein and HE protein project out of the viral envelope. In addition, 16 non-structural proteins (nsp1–16) have been identified in the genus Betacoronavirus [16,17]. Like other enveloped viruses, BCoV is sensitive to detergents and lipid-soluble solvents (including ether and chloroform) and is easily inactivated by most conventional disinfectants, formalin, and heat. The structural domain division of the BCoV genome and spike protein is intuitively shown in Figure 2, among which the N-terminal domain (NTD) and receptor-binding domain (RBD) regions in the S1 subunit and the S1/S2 cleavage site are the core hotspots of amino acid variation.

Coronaviruses demonstrate a high capacity to adapt to new environments through mutation and recombination, particularly when specific mutations occur in the S protein binding site. This ability allows the virus to effectively alter its host range and tissue tropism, while significantly influencing post-infection pathogenicity, transmissibility, and immune responses [18]. Therefore, understanding the clinical characteristics of emerging coronavirus variants and studying the evolutionary patterns of viral mutations can provide new insights into predicting the emergence of future variants and identifying key neutralizing antibody binding sites. This is crucial for controlling disease transmission and preventing future outbreaks.

## 3. Genetic Variations in Key Structural Proteins and Their Functional Implications

### 3.1. Variants in the Spike (S) Protein Gene

Variations in the host range and tissue tropism of coronaviruses are attributed to the spike (S) protein [19]. The S gene encoding the S protein is 4092 bp in length and encodes 1363 amino acids. This protein is cleaved by intracellular proteases into two functional domains [19]: the S1 subunit and the S2 subunit. The S1 subunit is located on the periphery and is responsible for recognizing and binding to host cell receptors [20]; it can induce the production of neutralizing antibodies [21] and hemagglutination activity [22]. The S1 sequence is variable, and mutations in this region may alter the virus’s antigenicity and pathogenicity [23,24]. From the perspective of structure-function coupling, even minor variations in the S1 region can cause local conformational changes in the receptor-binding interface, thereby affecting the virus’s efficiency of adsorption to and ability to invade host cells. At the same time, S1 is one of the primary regions recognized by host neutralizing antibodies; therefore, mutations in this region often carry dual implications of receptor adaptation and antigenic drift. In contrast, the S2 subunit primarily performs more fundamental functions such as membrane fusion and is subject to stronger structural constraints. Its high conservation suggests that the S1 subunit is more likely to serve as the key molecular window through which BCoV continuously adapts to the host environment and undergoes phenotypic differentiation. The S2 subunit is more conserved and is primarily responsible for mediating fusion between the cell membrane and the virus [25]. Studies have shown that the cleavage and activation of the coronavirus S protein play a crucial role in viral entry and virulence, and the S1 subunit is frequently used as a molecular epidemiological target for studying BCoV infection [26].

The neutralizing epitopes of BCoV S protein are mainly concentrated in the S1 subunit, which can be divided into two core antigenic regions according to structural and functional characteristics: the N-terminal domain (S1A region, NTD) with sialic acid binding activity and the C-terminal receptor-binding domain (S1B region, RBD) [15,27].The NTD contains multiple hemagglutination-related antigenic sites, which are the main targets of strain-specific neutralizing antibodies and show high amino acid polymorphism among different genotypes; the RBD mediates viral receptor recognition and cell adsorption, and is a key region for inducing cross-protective neutralizing antibodies. Driven by host immune pressure, the above two regions have the highest frequency of amino acid variation in the S protein, which is closely related to BCoV antigenic drift and immune evasion [21]. Studies have confirmed that mutations in the S gene sequence are the primary drivers of changes in viral biological phenotypes; however, the specific key amino acid mutations that determine virulence and antigenicity differences in BCoV have not yet been fully identified. This suggests that the relationship between S gene variants and viral phenotype is not determined by a single site, but is more likely a complex process involving the synergistic action of multiple sites and influenced by the overall protein conformation. In other words, a given amino acid substitution may only manifest significant changes in virulence, tropism, or antigenicity under specific genetic contexts. Therefore, future identification of key mutations should not be limited to describing sequence differences; it should also integrate structural predictions, receptor-binding analyses, and functional validation methods to gradually establish a continuous interpretive framework linking “mutation sites—protein structure—biological function—clinical phenotype.”

### 3.2. Hemagglutinin Esterase (HE) Gene Variants

The HE protein is a unique membrane protein found in bovine coronaviruses that forms spike-like projections on the viral surface; some β-coronaviruses also possess spike-like HE proteins. The HE protein consists of two 65 kDa glycosylated subunits that form a 140 kDa dimer via disulfide bonds and belongs to the class I transmembrane proteins. The HE protein contains lectin and esterase domains [28,29]. The lectin domain and esterase domain play important roles in viral entry and release [30]. The lectin domain possesses hemagglutination activity and receptor-binding activity; it can agglutinate mouse erythrocytes and bind to sialic acid receptors on host cells. Therefore, the HE protein is regarded as the second attachment protein responsible for initiating infection, in addition to the S protein [31,32]. The esterase domain possesses acetylesterase activity, which can inactivate receptor enzymes, remove receptors from the cell surface, and facilitate viral release, thereby accelerating the production of progeny viruses [30]. It is worth noting that the role of the HE protein during infection is not isolated; rather, it works in concert with the S protein to maintain the dynamic “attachment-detachment” equilibrium between the virus and the host cell surface. The S protein primarily mediates high-efficiency receptor recognition and membrane fusion for entry, while the HE protein regulates the intensity and duration of the interaction between the virus and the receptor through its lectin and esterase activities. When HE function changes, the virus’s retention, diffusion, and release rhythms on the cell surface may all be altered, thereby further affecting its tissue adaptability and transmission efficiency.

Mutations in the HE protein may affect the host’s receptor-binding function, thereby influencing the virus’s tissue tropism and host selection [33]. Studies have shown that the HE gene of human coronavirus OC43 (hCoV-OC43) has accumulated mutations during its adaptation to replication in the human respiratory tract, gradually losing its receptor-binding function [31]. The loss of receptor-binding function was accompanied by alterations in the receptor-cleaving activity mediated by the HE protein, and MHV underwent significant changes, with the receptor-specific binding site shifting from 9-O-Ac-Sia to 4-O-Ac-Sia [32]. Therefore, the evolution of the HE protein receptor may influence the host specificity and tissue tropism of BCoV, which is crucial for understanding zoonotic potential, virus–host adaptation, and clinical disease. Furthermore, receptor identification can provide insights for the development of antiviral drugs, vaccines, and diagnostic tests, which have significant implications for both human and animal health.

### 3.3. Variations in Other Proteins

Compared to S and HE, the nucleocapsid (N) gene is generally more conserved, as its primary function is to encapsidate the viral genomic RNA to form a ribonucleoprotein complex, which is subject to strong structural constraints. However, there are also some variable sites on the N protein; these variations may affect RNA-binding efficiency, protein dimerization, or interactions with host cell proteins, thereby indirectly influencing viral replication efficiency or virulence. Research on variants of the envelope protein (E) and membrane protein (M) is relatively scarce. Existing data suggest that these proteins are generally conserved; however, minor variations in certain transmembrane regions or the extracellular loop of the M protein may also affect viral assembly and budding.

In addition, variants in the coding regions of accessory proteins also warrant attention. Although these proteins do not typically constitute the core structure of viral particles, they may play important roles in regulating replication efficiency, influencing host cell responses, and participating in host adaptation. In particular, variants associated with ORF4 exhibit a certain degree of instability across different strains, suggesting that while they may not be absolutely essential for basic viral replication, they are potentially linked to a strain’s ecological adaptation, transmission efficiency, and phenotypic differences. Therefore, when interpreting the pathogenicity and epidemiological characteristics of different BCoV strains, auxiliary proteins should be incorporated into the overall variant analysis framework, in addition to focusing on surface proteins such as S and HE.

## 4. Analysis of the Association Between Genetic Variants and Clinical Manifestations and Tissue Tropism

Traditionally, bovine coronavirus (BCoV) has been regarded primarily as an intestinal pathogen capable of causing diarrhea in calves and winter diarrhea in adult cattle. However, since the 1990s, there have been increasing reports of BCoV strains isolated from cattle with respiratory diseases, confirming that the virus exhibits distinct tropism for respiratory tissues. Currently, there remains controversy regarding the molecular determinants of the shift in tropism between enteric BCoV (EBCoV) and respiratory BCoV (RBCoV). Several studies on coronaviruses have indicated that mutations in the S gene are the key region mediating changes in tissue tropism [34]. Some studies suggest that, over the course of long-term evolution, BCoV has gradually evolved from a single-tropic intestinal pathogen into a virus strain with dual intestinal and respiratory tropism [35]. Therefore, a systematic analysis of the association between variant amino acid sites and tissue tropism still requires more systematic sequence alignment and functional validation based on temporal sequences.

Based on existing research, scholars have identified a number of key S-protein mutation sites that may influence BCoV tissue tropism [3,36,37,38,39,40,41]. However, the direct causal relationship between these sites and tropism switching must still be rigorously validated using reverse genetics or pseudovirus systems before it can be definitively established. It should be noted that the establishment of tissue tropism does not imply that the virus is restricted to replicating in a single tissue within the host; rather, it is more likely to manifest as a redistribution of replication preferences across different tissues. Some strains, even when primarily causing intestinal symptoms, may simultaneously replicate and shed in the respiratory tract; conversely, respiratory-origin strains may not completely lose their ability to adapt to the intestine. It is precisely this phenotypic overlap that renders a simple dichotomization of strains based on sample origin or clinical symptoms insufficient, and suggests that the conversion of BCoV tissue tropism is more likely the result of the combined effects of multi-site synergistic mutations and the host environment. Overall, the S gene plays a central role in viral entry; mutations in its key regions not only alter the virus’s ability to recognize and bind to host cells but also influence its replication efficiency, pathogenicity, and immune evasion capabilities in different tissues, thereby leading to distinct clinical manifestations and tissue tropism.

Notably, the above-mentioned mutation sites are all candidate loci identified based on bioinformatics sequence alignment and correlation analysis between enteric and respiratory BCoV strains. Their direct causal relationship with tissue tropism shift has not been fully confirmed by functional experiments. Existing functional studies show that amino acid substitutions in the NTD sialic acid-binding region can alter the binding efficiency of the virus to 9-O-acetylated sialic acid receptors distributed in different tissues, which may be an important molecular basis for the dual tropism of BCoV to the intestinal and respiratory tracts [3,33]. In addition, synergistic mutations in the lectin domain and esterase domain of the HE protein can regulate the attachment and release efficiency of the virus on the cell surface, and jointly participate in the adaptation of tissue tropism with the S protein. To date, no definitive single core mutation that can independently drive the complete shift in BCoV tissue tropism has been identified. This phenotypic change is more likely to be the result of the synergistic effect of multiple sites in the S protein and HE protein, which needs to be further verified by reverse genetics systems and animal infection models. A thorough elucidation of the intrinsic relationship between mutations and clinical manifestations and tissue tropism holds significant theoretical and practical value for revealing the evolutionary patterns of BCoV and optimizing diagnostic methods and vaccine design.

## 5. Molecular Epidemiology and Phylogenetic Patterns of BCoV

### 5.1. Molecular Epidemiology of BCoV

Phylogenetic analyses based on the whole genome or key genes (such as the S gene) indicate that global bovine coronavirus (BCoV) strains can be clearly divided into two major genotypes: the Classical genotype (also known as the European genotype, EU-type) and the American-wild ruminant genotype (also known as the American genotype, AM-type) [41,42]. Although early studies suggested genetic differences between the American and European types [42], the genetic diversity landscape of these strains has become increasingly clear with the accumulation of epidemiological data based on the S gene and whole-genome sequences.

**Figure 3:**
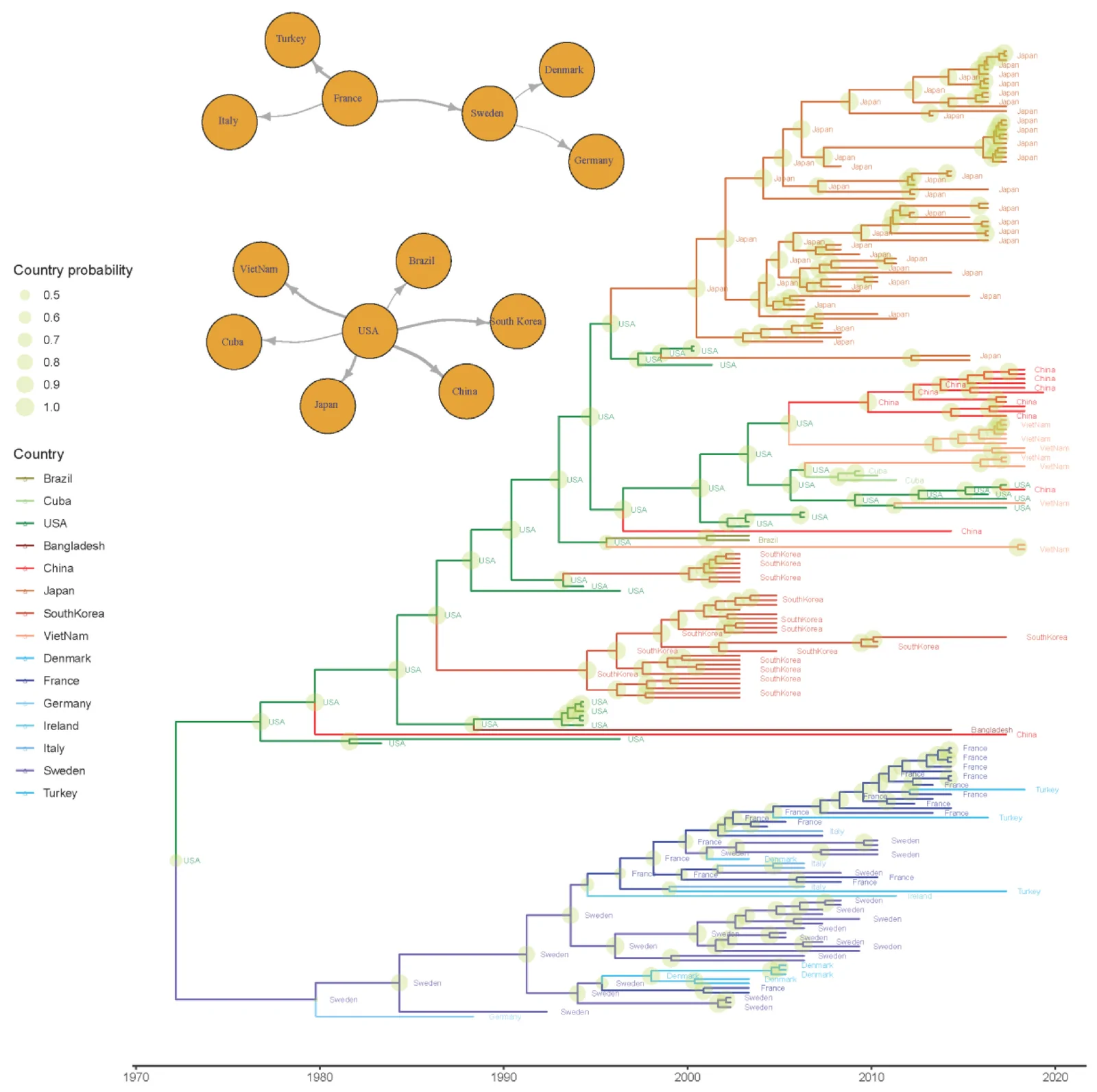
Time-scaled phylogenetic tree based on BoCV spike protein. The estimated ancestral geographic location has been color-coded (different nuances of green, red, and blue have been selected for American, Asian, and European countries), while the posterior probability is depicted as a circle on the corresponding node, whose size is proportional to the estimated value. The statistically supported migration routes have been represented in the upper left insert (the figure is cited from Franzo, G [43]).

Phylogenetic analysis revealed that several classic ancestral strains clustered into a single branch, while strains of European origin formed a distinct cluster, and strains from the United States and Asia converged into a single major evolutionary branch (Figure 3) [43]. A phylogenetic tree constructed based on the S gene further revealed that BCoV can be preliminarily classified into two major groups: GI and GII. In-depth analysis found that early classic strains from multiple countries (including the original Mebus strain and some Asian, American, and European strains) formed the GIa subgroup; European strains primarily constituted the GIb subgroup; and most American and Asian strains clustered into the GII group. Within this group, Korean strains form the independent GIIa subgroup, while some strains from the United States, Japan, and Vietnam, together with Chinese strains, constitute the GIIb subgroup. This phylogenetic structure further confirms the geographic clustering of BCoV evolution: Asian strains are more closely related to those from the United States, while European strains form an independent cluster. This distribution pattern may be closely related to frequent animal trade between the United States and Asia [44,45]. Notably, the phylogenetic tree shows that the S genes of respiratory BCoV (RBCoV) and enteric BCoV (EBCoV) do not form distinct clusters based on clinical presentation [46]. Furthermore, analysis indicates that BCoV strains tend to cluster according to the year or country in which they were detected or isolated, suggesting that the viruses may co-evolve through continuous exchange within their respective reservoirs. This result indicates that the molecular epidemiological pattern of BCoV exhibits both distinct temporal and spatial clustering. Strains from different regions do not evolve in complete isolation; rather, based on local sustained transmission, they are influenced by cross-regional cattle movements and trade activities, resulting in continuous new introductions and exports. The resulting phylogenetic structure retains the genetic characteristics of locally prevalent strains while continuously incorporating new genetic components, causing the global BCoV population to exhibit dynamic evolution rather than a static distribution.

Molecular tracing studies suggest that US-origin strains are the core group in the global transmission of BCoV, and have been introduced to Asia, South America and other regions along with international cattle trade, driving the formation of local epidemic lineages [43]. The widespread distribution of U.S. strains across all subclades in the phylogenetic tree supports this view, indicating that the evolution of U.S. BCoV strains may, to some extent, reflect the global evolutionary dynamics of the virus. In-depth analysis of the BCoV S protein revealed that, over time during viral evolution, gradual substitutions occurred at amino acid positions 146, 148, and 509: N146I, D148G, and N509T. These substitutions may lead to changes in protein structure. Among these, amino acids at positions 146 and 148 are located in the N-terminal domain (NTD) and its adjacent sialic acid-binding site region [27]; mutations at these sites may affect the structure of the glycan chains interacting with the NTD. The amino acid at position 509 is located in the C-terminal domain (CTD, i.e., the putative receptor-binding domain) and is also one of the sites in the S gene subject to positive selection pressure. Variations at these three sites were first observed in U.S. strains from 1996 to 1998, suggesting that BCoV may have undergone a significant phylogenetic evolution in the mid-to-late 1990s. Although these mutations were first identified in U.S. strains, they are all associated with the evolutionary transition from Group GI to Group GII and may therefore serve as important molecular markers for distinguishing between these two groups. Furthermore, studies have reported that amino acids at positions 146 and 148 may also differ between respiratory and intestinal strains [47], suggesting that the evolutionary relationships of American-type strains are equally significant for studying evolutionary characteristics associated with intestinal and respiratory tropism.

### 5.2. The Development and Evolution of BCoV

Coronaviruses exhibit high genetic plasticity and are particularly adept at adapting to new host environments, largely due to their strong genomic recombination capacity [13]. Analysis of available whole-genome sequences confirms a marked tendency toward recombination in bovine coronaviruses (BCoVs), with approximately 51% of sequences showing evidence of potential recombination events [43]. The frequent recombination events in BCoV may represent the molecular basis for changes in its tissue tropism and host specificity. Bioinformatic recombination detection requires careful interpretation, as multiple factors can influence its sensitivity and specificity [48,49]. Nevertheless, consistent evidence from multiple complementary analytical approaches, including RDP4, GARD, and phylogenetic network analysis, strongly supports the widespread occurrence of recombination in BCoV. This finding contrasts with earlier conclusions that recombination played only a marginal role in BCoV evolution [45]. Such discrepancy can be largely explained by differences in study design: previous investigations were limited by smaller sample sizes and analysis of individual genes or partial genomic regions, while the current evidence is derived from complete genome sequences with substantially improved analytical resolution. Although no single open reading frame shows a globally preferential recombination frequency, three statistically significant recombination hotspots have been identified along the genome of the reference Mebus strain, located at approximately position 4700 (within the pp1a coding region), position 21,600 (at the junction of the pp1ab 3′ end and the 32 kDa protein coding region), and position 23,900 (at the initiation region of the S gene). These hotspot regions have been verified at the individual gene level and are consistent with the results reported by Salem et al. (2020) [42], who identified a potential recombination event within the S gene. Notably, the presence of recombination hotspots in the pp1ab and S genes is not unique to BCoV, and similar patterns have been described in distantly related coronaviruses [50]. Since these genomic regions are functionally involved in viral replication efficiency and host cell attachment, and thus determine viral tropism and immunogenicity, recombination in these regions may confer fitness advantages to emerging recombinant strains, promoting their fixation, persistence, and spread in host populations [51,52].

Previous studies have also found that BCoV may have undergone recombination with other coronaviruses. For example, canine respiratory coronavirus CRCoV-k37 may have originated from a genetic recombination between BCoV and another canine coronavirus, CRCoV-BJ232 [53]. Meanwhile, human coronavirus HCoV-OC43, which is closely related to BCoV, may have originated from a recombination event between BCoV and porcine hemagglutinating encephalomyelitis virus. Compared to BCoV, the HCoV-OC43 genome exhibits a 290-nucleotide deletion (corresponding to the ns4.9 and ns4.8 coding regions of BCoV), a finding that supports an interspecies transmission event from cattle to humans [54]. Furthermore, genetic recombination and point mutations—particularly those occurring in the spike (S) gene—can facilitate the expansion of the virus’s host range [55]. From an evolutionary perspective, recombination and point mutations are not mutually exclusive processes but rather the two primary pathways driving genetic innovation in BCoV. The former can alter the combination of different gene modules within a short period, leading to relatively significant genetic changes in the virus; the latter, through continuous accumulation, enables fine-tuned adaptation to host immune pressure, tissue environments, and transmission conditions. The combined effect of these two processes allows BCoV to exhibit both gradual evolution and, at specific periods, more pronounced phylogenetic jumps.

The evolution of bovine coronavirus (BCoV) is a dynamic process driven by both high-frequency homologous recombination and sustained positive selection. Frequent genetic recombination is a key mechanism underlying its genetic diversity and expanding host range, while the spike (S) gene is subject to intense immune selection pressure, leading to continuous antigenic evolution. The analysis of the selective pressures acting on the S protein identified several sites under significant pervasive diversifying and purifying selection using different methods (Table 2). An even higher number of codons was detected under episodic diversifying selection with MEME (i.e., 4, 113, 155, 179, 220, 253, 257, 412, 499, 501, 509, 510, 546, 579, 717, 894, 1027, 1189, 1211, and 1298) [43]. These evolutionary forces have shaped a distinct global population structure, giving rise to two major genotypes: the European-centered “classical” type and the North American-origin “American” type, which has spread to Asia. The transmission pathways of these genotypes closely align with international trade networks.

## 6. Dynamics of Geographic Dispersal and Population Genetic Structure

### 6.1. Transmission of Intestinal BCoV

Since BCoV was first detected in the United States, BCoV-induced gastrointestinal infections have been reported on all five continents, with significant variations in incidence rates and patterns of circulation across different continents, countries, and regions. Prior to 2000, BCoV had already been confirmed to cause gastrointestinal diseases such as calf diarrhea and winter diarrhea in adult cattle in the Americas, Europe, and Asia. In the Americas, countries such as the United States, Canada, and Argentina reported higher positivity rates, ranging from 2.41% to 84%, suggesting that this region was an early and widespread area of BCoV prevalence [37,56,57,58,59,60]. In Europe, BCoV was detected in the United Kingdom in 1986 and in Belgium in 1999, with detection rates of 14% and 8%, respectively, indicating that enteric BCoV is also endemic in Europe [61,62].

Between 2000 and 2009, the prevalence of enteric BCoV was particularly pronounced in many countries across Asia and Europe. In Asia, the detection rates of BCoV in samples from calves with diarrhea ranged from 10.8% to 28.1% in Turkey and from 5.6% to 58.2% in South Korea, with the prevalence continuing to expand [34,35,38,39,40,63,64]. In Europe, the Netherlands and Italy also reported high detection rates of 2.80% and 46.74%, respectively [65,66]. In South America, Brazil recorded an intestinal BCoV detection rate as high as 68.6% during this period, indicating that intestinal BCoV can reach high levels of prevalence under intensive farming conditions [67,68,69,70].

Between 2010 and 2019, the distribution of intestinal BCoV further expanded to Oceania and Africa. BCoV was detected in calf fecal samples in Australia and New Zealand, with positivity rates of 21.6% and 14.0%, respectively [71,72]. In Africa, BCoV was also detected in intestinal samples from Algeria and Ghana, with positivity rates of 20.73% and 0.30%, respectively [73,74]. During the same period, epidemiological surveys were conducted in several Asian countries; BCoV was detected in fecal samples from calves with diarrhea in Iran, China, Thailand, India, and Vietnam, with positive rates of 7.2%, 12.20–69.05%, 12%, 8.88–16.00%, and 6.9%, respectively, confirming that intestinal BCoV is already widespread in major cattle-raising countries in Asia [75,76,77,78,79,80].

Overall, the spread of bovine coronavirus (BCoV) in the gut has shown a trend of expanding from localized outbreaks to widespread transmission and gradually becoming a global issue. The severity of outbreaks is closely linked to the level of intensive livestock farming and the frequency of cattle movement, and BCoV has now become a major pathogen causing gastrointestinal diseases in cattle worldwide.

### 6.2. Transmission of Respiratory BCoV

Since respiratory BCoV was first detected in respiratory samples in the United States, its role in bovine respiratory diseases has gradually become recognized, and reports of its prevalence worldwide have steadily increased [81]. In Japan became the first country in Asia to confirm BCoV positivity in nasal swab samples, marking the beginning of surveillance and detection of respiratory BCoV in the Asian region [44]. Between 2000 and 2009, respiratory BCoV infections were reported in several European countries. The positive detection rates in respiratory samples ranged from 9.60% to 65.85% in Italy and from 22.9% to 60.7% in Ireland, indicating that respiratory BCoV had established a stable prevalence in European cattle herds [82,83].

Between 2010 and 2019, respiratory BCoV further spread to Oceania. Australia detected BCoV in bovine respiratory samples for the first time, with a positivity rate ranging from 13.00% to 33.33%, thereby expanding the geographic distribution of this strain [84]. After 2020, BCoV was also detected in bovine respiratory samples in China’s Northeast region, with a positivity rate of 21.53%, confirming the presence and prevalence of respiratory BCoV infection in China’s cattle population [79]. To date, there have been no systematic reports of respiratory BCoV detection in African cattle herds, which may be related to the insufficient coverage of bovine respiratory pathogen surveillance in the region, rather than the absolute absence of respiratory BCoV infection.

**Figure 4:**
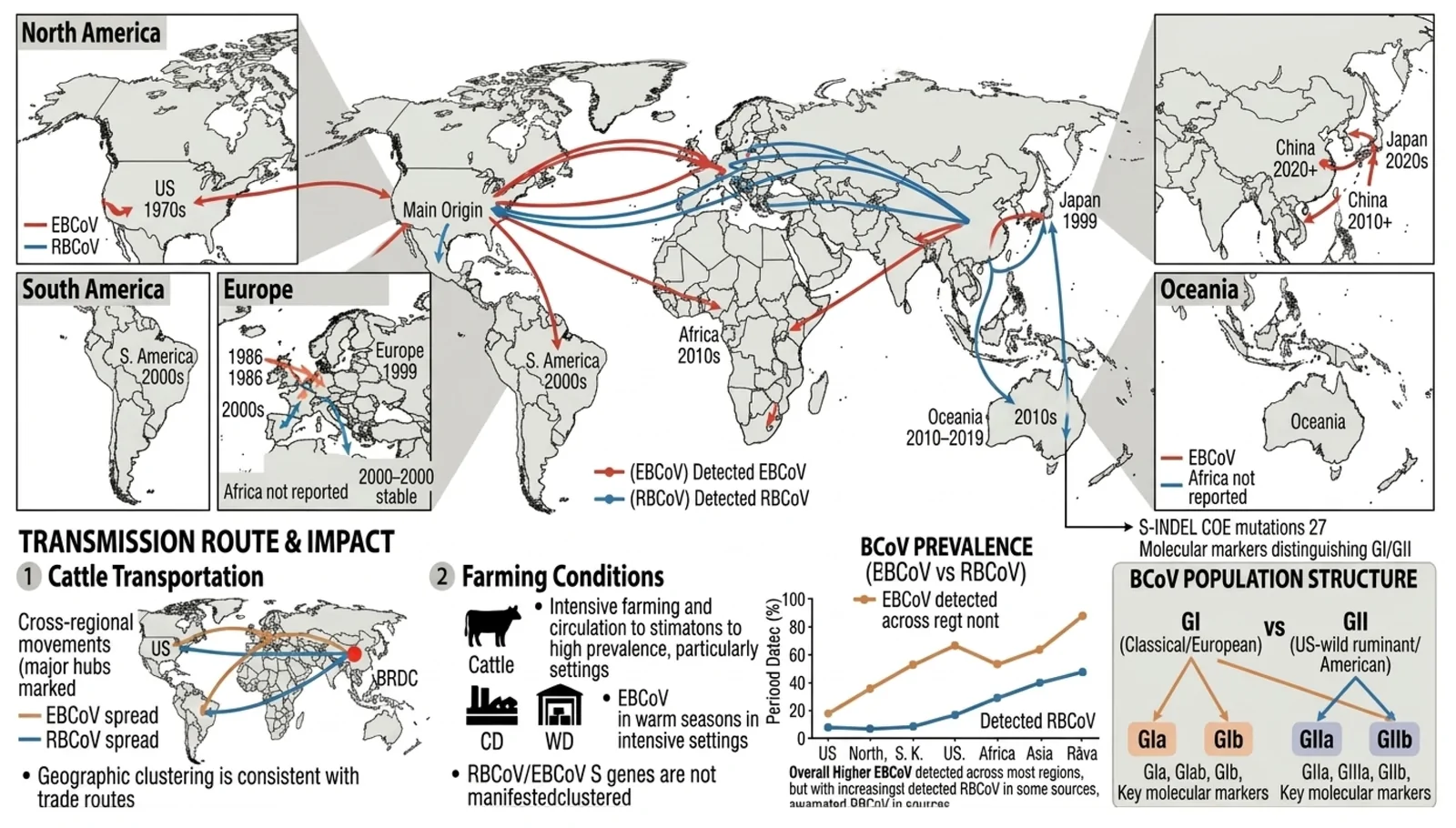
Geographic Spread and Population Structure of BCoV.

Overall, respiratory BCoV emerged later than enteric BCoV, and its transmission routes are similarly highly correlated with the interregional movement of live cattle and the concentrated circulation of livestock within farming facilities. With the expansion of surveillance coverage and improvements in detection technology, the detection rate of respiratory BCoV in bovine respiratory disease syndromes has been steadily increasing, making it a significant type that cannot be overlooked in global BCoV epidemiology and control. A comprehensive analysis of transmission data for both enteric and respiratory BCoVs reveals that their global spread is not merely a result of natural geographical expansion, but is closely linked to modern cattle farming practices, the frequency of live cattle movements, and the continuous improvement of surveillance systems. On the one hand, large-scale farming and high-density rearing create favorable conditions for the sustained circulation of the virus; on the other hand, differences among countries and regions in sample sources, testing methods, and surveillance intensity can also influence apparent comparisons of positivity rates and epidemiological patterns. Therefore, when interpreting geographical transmission dynamics, it is essential to focus not only on the genetic evolution of the virus itself but also to fully consider the combined influence of farming systems and surveillance biases on the formation of epidemiological data. The global geographic transmission and population structure of BCoV are shown in Figure 4.

## 7. The Potential Impact of Host Genetic Factors on BCoV Infection and Variation

To effectively enter host cells, viruses bind to specific cell surface receptor molecules [85,86]. The selection of entry receptors is governed by precise interactions that mediate both effective viral attachment to the cell surface and successful cellular infection. Viruses can exploit a wide range of cell surface molecules to penetrate host cells [87]; these molecules are major determinants of viral tropism and pathogenicity. Receptor-binding motifs in viruses are subject to evolutionary changes driven by immune surveillance, which can target key receptor-binding residues during the neutralization of viral infections. Consequently, it is common for viruses to evolve to use different cellular entry receptors during infection, or for related viruses to utilize different cell surface molecules to enter host cells [88]. This is the case with coronaviruses (CoV), whose use of different entry receptor molecules results in a broad host range and tissue tropism [9,19]. Some CoVs possess significant cross-species transmissibility, which is associated with the virus’s adaptation to use orthologous receptor molecules [18,89].

The host’s genetic background, particularly the polymorphism of receptor genes, directly influences the critical step of virus–receptor recognition, thereby significantly affecting the course of BCoV infection. Sequence variations in receptor genes among different individuals or populations alter the structure and expression characteristics of receptor molecules, leading to differences in viral binding and entry efficiency. These differences ultimately manifest as variations in susceptibility, infection rates, and the severity of clinical symptoms among individuals. At the same time, the differences in binding preferences resulting from host receptor gene polymorphisms exert continuous evolutionary pressure on the virus, driving adaptive mutations in BCoV’s receptor-binding regions to better adapt to host receptors and complete the cycle of infection and transmission. Thus, host genetic factors not only serve as intrinsic conditions influencing the onset and progression of BCoV infection but also, to a certain extent, contribute to driving viral variation and evolution; together, they form a crucial foundation for BCoV infection and its epidemiology. Furthermore, the role of host genetic factors is not necessarily limited to a binary distinction of “susceptibility or resistance,” but is more likely to manifest in continuous phenotypes such as infection thresholds, duration of viral shedding, local tissue replication levels, and the intensity of inflammatory responses. This implies that when the same viral strain enters cattle populations with different genetic backgrounds, it may exhibit varying transmission efficiencies and clinical outcomes. Consequently, incorporating host genetics into the BCoV research framework helps provide a more comprehensive explanation of epidemiological and pathogenic variations from the perspective of virus–host co-evolution.

## 8. Summary and Outlook: Challenges and Future Research Directions

In summary, this paper systematically reviews recent advances in the genetic variation and molecular epidemiology of bovine coronavirus (BCoV). It comprehensively elucidates BCoV as a major pathogen responsible for diarrhea in newborn calves, winter diarrhea in adult cattle, and bovine respiratory disease syndrome, noting that its genome exhibits the typical evolutionary characteristics of RNA viruses, including a high mutation rate and frequent homologous recombination. Studies indicate that the S1 subunit of the S gene, the HE gene, and ORF4—all accessory protein genes—constitute core mutation hotspots within the BCoV genome. Mutations and recombination in these regions directly mediate changes in viral antigenicity, shifts in tissue tropism, and immune evasion, serving as the key molecular basis for determining the pathogenicity, transmissibility, and epidemiological characteristics of viral strains. Among these, the S protein serves as a key structural component for receptor recognition, membrane fusion, and neutralizing antibody targeting. Its S1 subunit exhibits the most significant variation, with characteristic amino acid substitutions occurring in multiple neutralizing epitope regions, directly affecting viral entry efficiency and antigenic properties; The HE protein participates in regulating viral tissue tropism and the release process through synergistic changes in receptor-binding activity and esterase activity; structural genes such as N, E, and M are relatively conserved and can serve as stable and reliable targets for molecular diagnosis. Phylogenetic analysis confirms that global BCoV can be classified into two major genotypes: the classical (European) type and the American type. Strain evolution exhibits significant geographic clustering, closely aligning with transmission routes and international cattle trade flows, and has been subject to strong positive selection pressure during evolution, with mutations in the S protein antigenic sites being particularly prominent. Furthermore, the S genes of enteric and respiratory strains do not form distinct clusters based on clinical manifestations, suggesting that the switch in tissue tropism is determined by multi-site synergistic mutations. The host genetic background, particularly receptor gene polymorphisms, can directly influence viral entry efficiency and the course of infection, representing a critical component in the BCoV–host interaction that regulates the outcome of infection.

**Figure 5:**
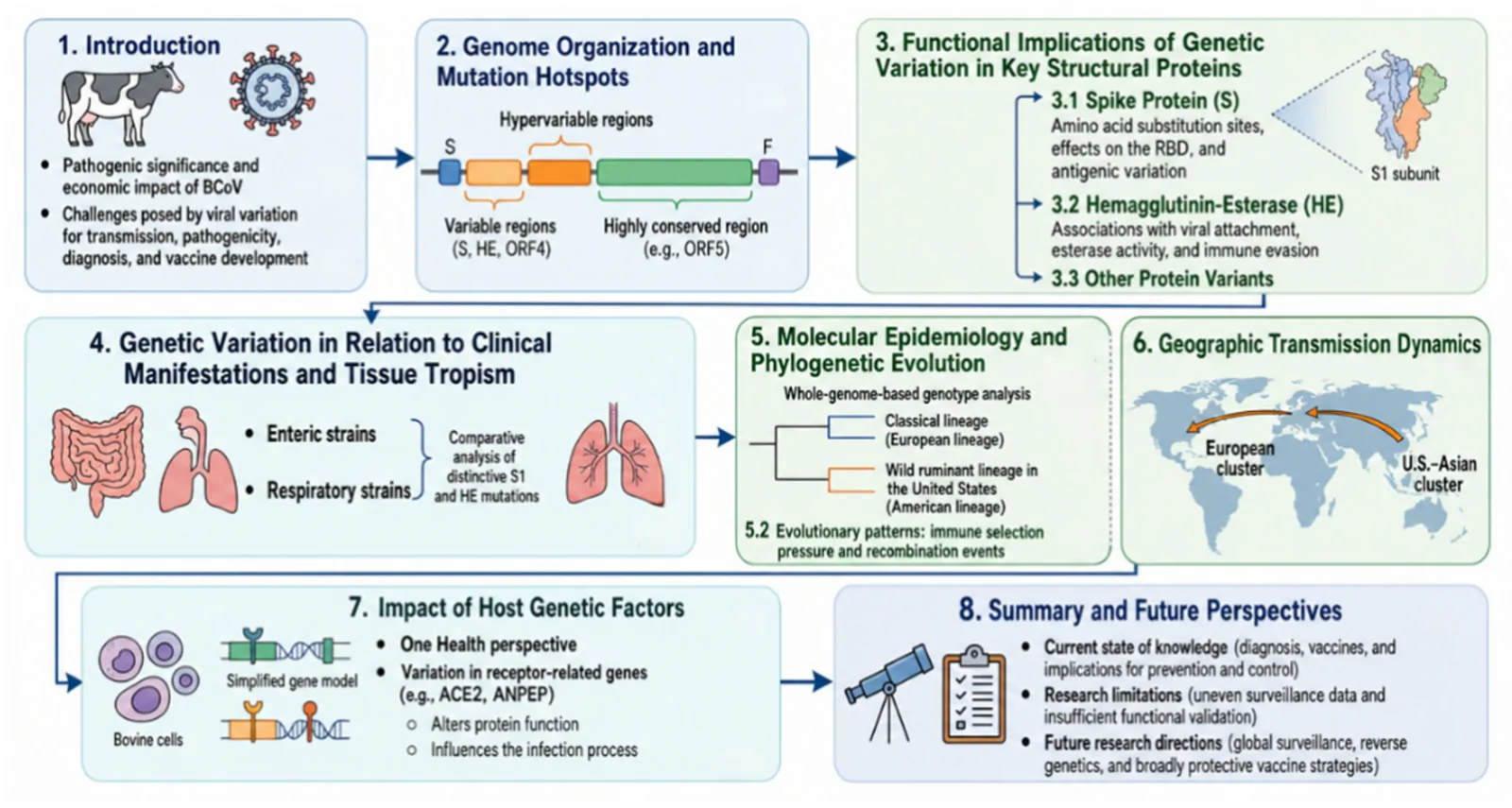
Overview of the analytical framework for BCoV genetic variation, covering genotype identification, mutation hotspot analysis, functional verification, and application scenarios of prevention and control.

Although significant progress has been made in current research on BCoV genetic variation, evolutionary patterns, and molecular epidemiology, several key scientific questions and practical shortcomings remain to be addressed. First, global genomic surveillance data is unevenly distributed; existing sequences are largely concentrated in North America, Europe, and East Asia, while data from major cattle-raising regions such as Africa, South America, and South Asia is scarce, making it difficult to fully characterize the virus’s global transmission network and long-term evolutionary dynamics. Second, the functional mechanisms of key variant sites remain unclear. Most inferences are based on sequence alignment and bioinformatics analysis, lacking direct functional validation through reverse genetics approaches; the causal relationships between variants and tissue tropism, pathogenicity, and immune evasion have not yet been fully elucidated. Third, existing diagnostic methods and commercial vaccines are largely based on early, classical strains, and their sensitivity for detecting currently circulating variants, as well as their cross-protective efficacy, require systematic evaluation. Fourth, the specific mechanisms by which host genetic factors regulate BCoV susceptibility and the course of infection remain unclear, and the association between receptor gene polymorphisms and clinical phenotypes lacks validation from large-scale population data. Overall, current BCoV research is gradually shifting from “identifying and describing variants” to “explaining how variants influence phenotypes and control efficacy.” This shift implies that future research must not only continue to accumulate sequence data but also strengthen the analysis of the biological significance of key variants, systematically integrating genomic information with structural and functional characteristics, clinical manifestations, host responses, and field epidemiological data. Only by establishing a complete chain of evidence—from genotype to phenotype and ultimately to control applications—can we truly enhance the scientific rigor of BCoV surveillance and early warning, diagnostic optimization, and vaccine updates. The overall analytical framework of BCoV genetic variation is presented in Figure 5.

Building on the existing research foundation and the practical prevention and control needs of the cattle industry, systematic and in-depth research could be conducted in the future in the following areas:

1. Continue to improve the BCoV molecular epidemiological surveillance network; expand whole-genome sequencing of strains from different regions and clinical types (gastrointestinal and respiratory); systematically track mutation trends, recombination events, and cross-regional transmission routes; and enrich and update the database of locally circulating strains.
2. Accelerate the development of a stable and efficient BCoV reverse genetics system. Perform site-directed mutagenesis and functional rescue experiments on key genes such as S and HE to precisely elucidate their specific roles in viral entry, replication, tissue tropism, and antigenic variation, thereby establishing clear correlations between genotype and phenotype.
3. Optimize detection technologies such as quantitative real-time PCR and ELISA by targeting highly conserved regions like the N gene to enhance the detection capability of variant strains; design broad-spectrum vaccines based on the S2 subunit and conserved antigenic epitopes to improve cross-protection against different genotypes of circulating strains.
4. Conduct an in-depth analysis of how host receptor gene and immune-related gene polymorphisms influence BCoV infection, replication, and pathogenesis; screen for disease-resistance-associated molecular markers to provide a genetic basis for disease-resistant breeding and precision prevention and control in cattle herds.
5. Conduct cross-neutralization tests between field strains and existing vaccine strains to systematically evaluate the immunoprotective efficacy of current vaccines, thereby providing data support for vaccine strain updates and the optimization of immunization protocols.

Through the aforementioned systematic research, we can further elucidate the mechanisms of BCoV mutation and evolution, the patterns of its transmission and prevalence, and its pathogenic mechanisms. This will comprehensively enhance the levels of disease surveillance and early warning, precise diagnosis, and comprehensive prevention and control, thereby providing a solid scientific basis for effectively reducing the economic losses caused by BCoV-related diseases and ensuring the healthy and sustainable development of the cattle industry.

## Figures and Tables

**Table 1:** Comparison of genetic diversity and evolutionary rate among typical coronaviruses.

Virus	Whole-Genome Nucleotide Diversity	Evolutionary Rate (Substitutions/Site/Year)
BCoV	0.5–2.0%	~10^−4^–10^−3^
PEDV	2.0–5.0%	~10^−3^
SARS-CoV-2	~1.0–3.0%	~10^−3^
MHV	1.0–3.0%	~10^−4^–10^−3^

**Table 2:** Codons of the spike protein detected under statistically significant diversifying selection using different methods.

	FEL		FUBAR		SLAC	
Position	dN-dS	*p*-Value	dN-dS	Posterior Probability	dN-dS	*p*-Value
113	4.714	0.011	7.060	0.993	5.711	0.013
174	-	-	-	-	4.000	0.039
179	9.127	0.030	17.724	0.994	10.129477	0.012
257	3.009	0.039	4.547	0.937	-	-
400	-	-	3.326	0.912	-	-
499	5.817	0.013	12.690	0.997	5.816	0.021
501	7.518	0.001	14.566	1.000	12.412	0.000
509	9.840	0.001	17.373	1.000	9.071	0.002
510	3.790	0.024	6.358	0.984	4.722	0.030
525	-	-	8.060	0.917	6.462	0.034
572	-	-	4.962	0.938	-	-
579	3.456	0.032	5.916	0.972	4.219	0.046
966	-	-	7.742	0.972	-	-
1298	1.307	0.026	1.403	0.918	-	-

## Data Availability

This is a review article, and no original experimental data have been generated. All information included is based on previously published studies that are appropriately referenced within the manuscript.
